# Modeling and Analysis of Mobility Management in Mobile Communication Networks

**DOI:** 10.1155/2014/250981

**Published:** 2014-05-05

**Authors:** Woon Min Baek, Ji Hyun Yoon, Chesoong Kim

**Affiliations:** ^1^Kongju National University High School, Kongju, Chungnam 314-801, Republic of Korea; ^2^Korean Minjok Leadership Academy, Hoengseong, Kangwon 225-823, Republic of Korea; ^3^Sangji University, Wonju, Kangwon 220-702, Republic of Korea

## Abstract

Many strategies have been proposed to reduce the mobility management cost in mobile communication networks. This paper studies the zone-based registration methods that have been adopted by most mobile communication networks. We focus on two special zone-based registration methods, called two-zone registration (2*Z*) and two-zone registration with implicit registration by outgoing calls (2*Zi*). We provide a new mathematical model to analyze the exact performance of 2*Z* and 2*Zi*. We also present various numerical results, to compare the performance of 2*Zi* with those of 2*Z* and one-zone registration (1*Z*), and show that 2*Zi* is superior to 2*Z* as well as 1*Z* in most cases.

## 1. Introduction

The number of mobile subscribers has been increasing, and the more accelerated growth of smart phone subscribers is expected with 4G networks. In a mobile communication network, since a mobile (mobile phone) is continually moving due to its basic characteristic, mobility management of the mobile is essential, to provide communication services with high quality.

One of the most important issues in mobility management is the location tracking. The location of a mobile must be maintained to connect an incoming call to the mobile if required. Location registration and paging are two basic functions to locate a mobile. Location registration is the series of processes to register a mobile's new location information in the system database, and paging is the series of processes to page the mobile in current location, to find mobile's exact cell and connect an incoming call, when an incoming call arrives. Since there is a tradeoff between location registration cost and paging cost, it is essential to analyze location registration cost and paging cost, in order to find the optimal location tracking method.

Various location registration methods have been proposed for mobile communication networks [1–9]. However, the most important location registration method is zone-based registration [5–8], since it is adopted by most mobile communication networks.

In this study, zone-based registration is considered. In zone-based registration, each mobile has a* zone_list*, where the visited zone is stored. If a mobile moves to a new zone, which is not in its current* zone_list*, the new zone is stored in the* zone_list*, and the mobile registers its new location information in the system database. A mobile may have more than one zone in zone-based registration [5], but most mobile communication networks adopt one-zone registration (1*Z*) because of ease of operation.

Lin [6] suggested a precise mathematical model, to analyze the performance of the case where a mobile has two zones, and compared the performance of the two cases, in which the number of zones is one and two. In addition, Jang et al. [7] considered implicit registration effects by outgoing calls, to improve the performance of the case where a mobile has two zones. However, Jang et al. [7] assumed a special mobility model and provided just a rough approximation of the performance.

Even Lin [6] provided a precise mathematical model for the performance of the case where a mobile has two zones, but his model is too complex to be applied to Jang et al.'s study [7], which considers implicit registration by outgoing calls under two registered zones, to improve the performance.

In this study, we derive a new mathematical model to analyze the exact performance of two-zone registration (2*Z*) and two-zone registration with implicit registration by outgoing calls (2*Zi*).  Section 2 briefly describes general zone-based registration methods.  Section 3 describes the mathematical model that we derived, to analyze the performance of 2*Z* and 2*Zi*.  Section 4 presents the computational results of the signaling cost on radio channels, using our model.  Section 5 summarizes the results and suggests future research directions.

## 2. Zone-Based Registration and 2*Z*

**Figure 1:**
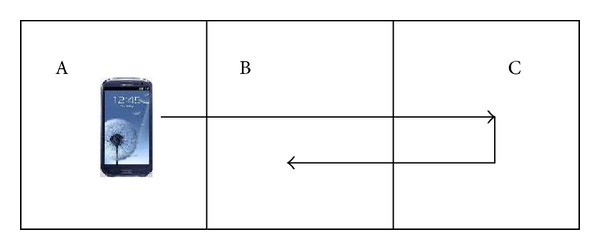
Movement of a mobile.

In zone-based registration, whenever a mobile moves to a new zone that is not in its current* zone_list*, this new zone is stored in its* zone_list*, and the mobile registers new location information in the system database. If a mobile can have only one registered zone (i.e., one-zone registration, 1*Z*), the mobile stores the newly entered zone in its* zone_list* every time it moves from one zone to another. Thus, the system knows the zone in which the mobile is located, and the paging process always meets with success for incoming calls.  Figure 1 shows the movement of a mobile.

However, if the mobile can have two registered zones (i.e., two-zone registration, 2*Z*), the system sometimes does not know the exact zone in which the mobile is located. For example, two zones, A and B, are stored in the* zone_list*, and the mobile is currently in zone B. Let the left-hand zone denote the most recently registered zone in the following* zone_list* and* system_DB*:   zone_listBA system_DBBA

When the mobile enters a new zone, C, then the* zone_list* and* system_DB* are changed as follows:zone_listCB system_DBCB

If an incoming call arrives in this situation, then the system pages the mobile in zone C and this paging succeeds (this is referred to as* zone hit*).

Consider another case. If the mobile reenters zone B, then* zone_list* is changed as below, but location registration does not occur, because zone B is already stored in the current* zone_list*:   zone_listBC system_DBCB

In this case, the system does not know the correct zone (B) in which the mobile is located, and the paging process is somewhat complicated. If an incoming call arrives, the system pages the mobile in zone C, since the mobile is known to be in this zone. If there is no response to paging after a predetermined time, the system recognizes that the mobile is not in zone C, but in zone B, and next pages the mobile in zone B (this is referred to as* zone miss*; note that the second paging always succeeds). This is one of the disadvantages of 2*Z*. That is, when the mobile reenters a previously visited zone and an incoming call occurs, the system must make two-step paging because of* zone miss*.

However, two-step paging can be avoided, if two-zone registration with implicit registration by outgoing calls (2*Zi*) is employed. For example, if the mobile enters zones in the order A→B→C→B and the outgoing call occurs in the last zone, B, then the call setup messages of an outgoing call can provide the system with the exact zone, B, and the system can successfully page the mobile in zone B at this time. In other words, when the mobile makes the outgoing call, call setup messages can provide the correct location information of the mobile implicitly, without an additional location registration message. This is termed implicit registration [5, 7].

Henceforth, for convenience, we refer to location registration by entering a new zone as regular location registration (RR) and the location registration effect by an outgoing call as implicit registration (IR).

2*Zi* was considered and analyzed by Jang et al. [7], assuming the following 4-direction mobility model.

- A mobile moves in a straight line, until it reaches a turning point.
- When it reaches the turning point, it can choose one of 4 directions with equal probability.
- The distance between two consecutive turning points is exponentially distributed.

When the above mobility model is assumed, it is impossible to express the exact equations for the performance measures such as registration cost and paging cost. Thus, for convenience, Jang et al. [7] assumed that, once a mobile enters a zone and makes one direction change, it is located at a random point in the zone. This wild assumption makes it possible to obtain some performance measures, but they are inherently rough approximations of the exact performance. Lin [6] provided a precise model for the performance of 2*Z*, but Lin's model is too complex to be applied to Jang et al.'s study, which considers implicit registration by outgoing calls under two registered zones.

## 3. New Mathematical Model and Performance Analysis

In this section, we propose a new mathematical model, to analyze the exact performance of 2*Z* and 2*Zi*. The radio channel is the most important resource determining the network performance in mobile communication networks. Although, thanks to technological enhancements, the capacity of mobile communication systems has been greatly improved, radio channels still have their own capacity and technological limit. Thus, the signaling cost on radio channels determines the performance of the entire mobile communication system. The performance analysis of 2*Z* and 2*Zi* is conducted from this viewpoint.

### 3.1. Notations and Assumptions

The following notations are defined, to analyze the signaling cost on radio channels:

 
* N*_1_(*Z*): number of location registrations between two incoming calls in the location registration method *Z*;
 
* C*_1_(*Z*): total location registration cost between two incoming calls in the location registration method *Z*;
 
* N*_2_(*Z*): number of cells for paging for an incoming call in the location registration method *Z*;
 
* C*_2_(*Z*): total paging cost per zone for an incoming call in the location registration method *Z*;
 
* C*_*p*_: signaling cost for paging per cell on radio channels;
 
* C*_*u*_: signaling cost for one location registration on radio channels;
 
* θ*: probability of returning to the registered zone;
 
* n*: number of cells per zone;
 
* t*_*c*_: interval between two incoming calls (r. v.);
 
* t*_*oc*_: interval between two outgoing calls (r. v.);
 
* t*_*m*_: sojourn time in a zone (r. v.);
 
* λ*_*c*_: arrival rate of incoming calls;
 
* λ*_*oc*_: arrival rate of outgoing calls;
 
* *1/*λ*_*m*_: mean of sojourn time in a zone;
 
* ρ*: call-to-mobility ratio (CMR), *ρ* = *λ*_*c*_/*λ*_*m*_;
 
* f*_m_*(*s*): Laplace-Stieltjes Transform for  *t*_*m*_ ( = ∫_*t*=0_^*∞*^ *e* ^−*st*^ *f*_*m*_(*t*)*dt*);
 
* *Λ: probability that an outgoing call occurs before an incoming call, Λ = Pr⁡[*t*_*oc*_ ≤ *t*_*c*_] = *λ*_*oc*_/(*λ*_*c*_ + *λ*_*oc*_);
 
* p*: probability that an outgoing call occurs while in the zone, *p* = Pr⁡[*t*_*oc*_ ≤ *t*_*m*_] = (*λ*_*m*_/*λ*_*oc*_)[1 − *f*_m_*(*λ*_*oc*_)];
 
* q*: probability that an outgoing call does not occur while in the zone, *q* = 1 − *p*.

In addition, the following assumptions are necessary to analyze the signaling cost on radio channels:

- the incoming calls to a mobile form a Poisson process with *λ*_*c*_;
- the outgoing calls from a mobile form a Poisson process with *λ*_*oc*_;
- the sojourn time in a zone follows a general distribution with a mean of 1/*λ*_*m*_;
- the first paging is applied to the most recently registered zone. If there is no response, the second paging is applied to the other zone.

### 3.2. Performance Analysis of 2*Z* and 2*Zi*

This section studies the performance analysis for 2*Z* and 2*Zi*. We first estimate registration cost and paging cost between two incoming calls, which constitute the total signaling cost.

To find registration cost and paging cost between two incoming calls, let us introduce the probability *α*(*K*) that the mobile moves across *K* zones between two incoming calls. We use Lin's result on *α*(*K*) [6], because this is closely related to our study:

(1) $$\begin{array}{c} \alpha \left(K\right)=\{\begin{array}{cc} 1-\frac{1}{\rho }\left[1-f_{m}^{*}\left(\lambda_{c}\right)\right], & K=0,\\ \frac{1}{\rho }{\left[1-f_{m}^{*}\left(\lambda_{c}\right)\right]}^{2}{\left[f_{m}^{*}\left(\lambda_{c}\right)\right]}^{K-1}, & K\geq 1. \end{array} \end{array}$$

#### 3.2.1. Registration Cost

The number of RRs between two incoming calls is [6]

(2) $$\begin{array}{c} N_{1}\left(2Z\right)=\overset{\infty }{\underset{k=0}{\sum }}\;\overset{K}{\underset{i=0}{\sum }}i\left(\begin{array}{c} k\\ i \end{array}\right)\theta^{k-i}{\left(1-\theta \right)}^{i}\alpha \left(k\right)=\frac{\left(1-\theta \right)}{\rho }. \end{array}$$

The number of RRs between two incoming calls in 2*Zi* is the same as that in 2*Z*:

(3) N1(2Zi)=(1−θ)ρ.

Thus, the location registration cost between two incoming calls is

(4) C1(2Z)=C1(2Zi)=(1−θ)Cuρ.

#### 3.2.2. Paging Cost

Next, consider the paging cost. The paging cost can be derived by multiplying the number of cells to page by the paging cost per cell. In the case of 2*Z* and 2*Zi*, the number of cells to page for an incoming call is the sum of the cases where the system has correct location information (first paging success or* zone hit*) and incorrect location information (first paging failure or* zone miss*):

(5) N2(2Z)=n+n·Pr⁡(zone  miss  in  2Z)=n+n[1−Pr⁡(zone  hit  in  2Z)],N2(2Zi)=n+n[1−Pr⁡(zone  hit  in  2Zi)].

To obtain the probability of the first paging success in (5), let us define the conditional probability *S*_*Z*_(*K*) that, given that the mobile moves across *K* zones between two incoming calls, the first page succeeds in the location registration method* Z*. Then, the probabilities that the first page succeeds in 2*Z* and 2*Zi* are, respectively,

(6) $$\begin{array}{c} \text{ Pr}\left[\text{zone hit in}\;\;2Z\right]=\overset{\infty }{\underset{K=0}{\sum }}S_{2Z}\left(K\right)\alpha \left(K\right),\\ \text{Pr}\left[\text{zone hit in}\;\;2Zi\right]=\overset{\infty }{\underset{K=0}{\sum }}S_{2Zi}\left(K\right)\alpha \left(K\right). \end{array}$$

To obtain the above probabilities, we need to derive the general expressions of *S*_2*Z*_(*K*)  and  *S*_2*Zi*_(*K*).

For the sake of convenience, we first derive the probability *S*_2*Zi*_(*K*) that the first page succeeds in 2*Zi*, given that the mobile moves across *K* zones between two incoming calls.

*(1) Derivation of the Conditional Probability* *S*_2*Zi*_(*K*). Let us consider the following sequential procedure for deriving the general expression of *S*_2*Zi*_(*K*).

(i) For *K* = 0, no movement occurs between two incoming calls, so the first page always succeeds, and *S*_2*Zi*_(0) = 1.
(ii) For *K* = 1, the probability that the first page succeeds can be obtained in the following two cases.
  (1) Case 1. The mobile moves to a new zone, with probability (1 − *θ*). In this case, the* zone_list* is updated by RR; thus, the probability that the first page succeeds is the same as that when *K* = 0.
  (2) Case 2. The mobile moves back to the zone from whence it came, with probability *θ*. In this case, the system has incorrect information as to which zone the mobile is located in, but the* zone_list* can be updated by IR, with probability Λ that an outgoing call occurs before an incoming call. Therefore,
    (7) $$\begin{array}{c} S_{2Zi}\left(1\right)=\left(1-\theta \right)S_{2Zi}\left(0\right)+\theta \Lambda . \end{array}$$
(iii) For *K* = 2, the probability that the first page succeeds can also be obtained in the following two cases.
  (1) Case 1. The first movement of the mobile is to a new zone, with probability (1 − *θ*). In this case, the* zone_list* is updated; thus, the probability that the first page succeeds is the same as that when *K* = 1.
  (2) Case 2. The first movement of the mobile is back to the zone from whence it came, with probability *θ*. In this case, if the* zone_list* is updated by IR, with probability* p* that an outgoing call occurs while the mobile is in the zone, then the probability that the first page succeeds is the same as that when *K* = 1. If an outgoing call does not occur while the mobile is in the zone, then the system has incorrect location information, and the probability that the first page succeeds is the same as that when *K* = 0, because the system will have correct location information when the mobile either enters a new zone or moves back to the zone from whence it came, in the second movement. Therefore,
    (8) $$\begin{array}{c} S_{2Zi}\left(2\right)=\left(1-\theta \right)S_{2Zi}\left(1\right)+\theta \left[pS_{2Zi}\left(1\right)+\left(1-p\right)S_{2Zi}\left(0\right)\right]. \end{array}$$
(iv) The process can be generalized, when *K* = *k*. In the first movement after an incoming call, the mobile moves to a new zone with probability (1 − *θ*) or moves back to the zone from whence it came, with probability *θ*.
  (1) Case 1. In the case where the mobile moves to a new zone, the probability that the first page succeeds is the same as that when *K* = *k* − 1.
  (2) Case 2. In the case where the mobile moves back to the zone from whence it came, if an outgoing call occurs while the mobile is in the zone with probability* p*, then the* zone_list* is updated by IR, and the probability that the first page succeeds is the same as that when *K* = *k* − 1. Otherwise, the system will have the correct location information after the mobile makes one more movement, by either entering a new zone or moving back to the zone from whence it came. Thus, the probability that the first page succeeds is the same as that when *K* = *k* − 2. Therefore, we get a recurrence formula for *S*_2*Zi*_(*k*) as follows:
    (9) S2Zi(k)=(1−θ)S2Zi(k−1) +θ[pS2Zi(k−1)+(1−p)S2Zi(k−2)],for  k=2,3,…,S2Zi(0)=1,  S2Zi(1)=(1−θ)+θΛ.

Note that Λ = 0 and *p* = 0 in the case of 2*Z*, because IR by an outgoing call is not employed. Therefore,

(10) S2Z(k)=(1−θ)S2Z(k−1)+θS2Z(k−2),for  k=2,3,…,S2Z(0)=1,  S2Z(1)=(1−θ).

*(2) Paging Cost for an Incoming Call.* Using (9) and (10), for 2*Z* and 2*Zi,* respectively, (5) can be written by

(11) $$\begin{array}{c} N_{2}\left(2Z\right)=n+n\left(1-\overset{\infty }{\underset{K=0}{\sum }}S_{2Z}\left(K\right)\alpha \left(K\right)\right),\\ N_{2}\left(2Zi\right)=n+n\left(1-\overset{\infty }{\underset{K=0}{\sum }}S_{2Zi}\left(K\right)\alpha \left(K\right)\right). \end{array}$$

Using the above results, we can compute the number of cells required when an incoming call occurs, and we can find the paging cost, by multiplying this number by the paging cost per cell. Finally, the total paging costs for an incoming call for 2*Z* and 2*Zi* are, respectively,

(12) $$\begin{array}{c} C_{2}\left(2Z\right)=\left[n+n\left(1-\overset{\infty }{\underset{K=0}{\sum }}S_{2Z}\left(K\right)\alpha \left(K\right)\right)\right]C_{p},\\ C_{2}\left(2Zi\right)=\left[n+n\left(1-\overset{\infty }{\underset{K=0}{\sum }}S_{2Zi}\left(K\right)\alpha \left(K\right)\right)\right]C_{p}. \end{array}$$

#### 3.2.3. Total Signaling Cost

The total signaling cost on radio channels is derived by combining the registration cost and the paging cost as follows:

(13) C(2Z)=(1−θ)Cuρ +[n+n(1−∑K=0∞S2Z(K)α(K))]Cp,C(2Zi)=(1−θ)Cuρ +[n+n(1−∑K=0∞S2Zi(K)α(K))]Cp.

#### 3.2.4. Propositions for Explicit Expressions of Costs

**Proposition 1**

The general solution of (9) is

(14) $$\begin{array}{c} S_{2Zi}\left(n\right)=1+\frac{-\theta \left(1-\Lambda \right)\left[1-{\left(-\theta q\right)}^{n}\right]}{1+\theta q}. \end{array}$$

**Proof**

For convenience, let us omit subscripts. Rearranging the above equation, we can get

(15) S(n)−S(n−1)=−θq[S(n−1)−S(n−2)],for n=2,3,….

It can be seen that differences of the progression form a geometric progression with equal ratio (−*θq*). Then, the general term of the progression *S*(*n*)  can be easily obtained as follows:

(16) S(n)=S(0)+∑i=1n(−θq)i=1+−θ(1−Λ)[1−(−θq)n]1+θq={1+−θ(1−Λ)[1−(θq)n]1+θqif  n  is  even  number1+−θ(1−Λ)[1+(θq)n]1+θqif  n  is  odd  number.

**Proposition 2**

The general solution of (10) is  *S*_2*ZR*_(*n*) = [1 − (−*θ*)^*n*+1^]/(1 + *θ*).

**Proof**

For convenience, let us omit subscripts. In the case of 2Z, since IR by an outgoing call is not employed, Λ = 0 and *p* = 0. Inserting these values into (10), we have

(17) S(n)=[1−(−θ)n+1]1+θ={1+θn+11+θif  n  is  even  number 1−θn+11+θif  n  is  odd  number.

**Proposition 3**

Equation (17) gives the same *P*[*zone* *hit* *in*  2*Z*] as in Lin's study [6].

**Proof**

From (18), (19), and (20) of Lin's study [6],

(18) P[zone  hit  in  2Z]=ω1+ω2+ω3=α(0)+∑K=1∞ω2(K)α(K)+∑i=1∞θ2iα(2i)=α(0)+∑K=1∞1−θ2⌊(K−1)/2⌋+21+θα(K) +∑i=1∞θ2iα(2i).

If we express the previous equation as ∑_*i*=1_^*∞*^ *z*(*K*)*α*(*K*),   it is easy to show that *z*(*K*)  is as follows:

(19) z(K)  ={1,if  K=0,1−θK1+θ+θK=1+θK+11+θ,if  K  is  even  number, 1−θK+11+θ+0=  1−θK+11+θ,if  K  is  odd  number.

Since *z*(*K*) = *S*(*K*)  for all  *K* ≥ 0, the proof is complete.

Note that the above proposition implies that our model includes Lin's model on 2Z [6].

As shown in the appendix, the probability that the first paging succeeds in 2Zi, given that the mobile moves across *k* zones between two incoming calls, is composed of three probabilities for three exclusive cases, *ω*_1_(*k*),  *ω*_2_(*k*), and  *ω*_3_(*k*),   and their sum, *ω*(*k*), is

(20) ω(k) ={(1−θ)(1−Λ)[1−θk+1qk+1]1−θ2q2  +  p(1−Λ)θ2q[1−θk−1qk−1]1−θ2q2+Λ,    if  K  is  odd  number  (k≥3),(1−θ)(1−Λ)[1−θkqk]1−θ2q2+θkqk(1−Λ)  +  p(1−Λ)θ2q[1−θkqk]1−θ2q2+Λ,    if  K  is  even  number  (k≥2),(1−θ)(1−Λ)+Λ=1−θ+θΛ,    if  k=1,1,    if  k=0.

**Proposition 4**

Equation (14),  *S*(*k*) = 1 + (−*θ*(1 − Λ)[1 − (−*θq*)^*k*^]/(1 + *θq*)), is the same as (20).

**Proof**

(i) When *k* = 0 and *k* = 1, it is trivial.

(ii) When *k* is even number (*k* ≥ 2),

(21) ω(k)=(1−θ)(1−Λ)[1−θkqk]1−θ2q2+θkqk(1−Λ) +p(1−Λ)θ2q[1−θkqk]1−θ2q2+Λ=(1−θ)(1−Λ)[1+θ2q2+θ4q4+⋯+θk−2qk−2] +θkqk(1−Λ)+p(1−Λ)θ2q ×[1+θ2q2+θ4q4+⋯+θk−2qk−2]+Λ=1−θ(1−Λ)[1−θq+θ2q2−θ3q3+θ4q4+⋯       +  θk−2qk−2−θk−1qk−1]1+θq1+θq=1−θ(1−Λ)[1−θkqk]1+θq.

(iii) When *k* is odd number (*k* ≥ 3),

(22) ω(k)=(1−θ)(1−Λ)[1−θk+1qk+1]1−θ2q2+p(1−Λ)θ2q ×[1−θk−1qk−1]1−θ2q2+Λ=1−θ(1−Λ)[1−θq+θ2q2−θ3q3+θ4q4+⋯       −  θk−2qk−2+θk−1qk−1]1+θq1+θq=1−θ(1−Λ)[1+θkqk]1+θq.

**Proposition 5**

The explicit expressions of *C*(2*Z*) and *C*(2*Zi*) are

(23) $$\begin{array}{c} C\left(2Z\right)=\frac{\left(1-\theta \right)C_{u}}{\rho }+\left[n+n\left(\frac{\theta \left[1-f_{m}^{*}\left(\lambda_{c}\right)\right]}{\rho \left[1+\theta f_{m}^{*}\left(\lambda_{c}\right)\right]}\right)\right]C_{p}, \end{array}$$

(24) C(2Zi)=(1−θ)Cuρ +[n+n(θ[1−fm∗(λc)](1−Λ)(1+θq)ρ(1+θq)[1+θqfm∗(λc)])]Cp.

**Proof**

The result follows from

(25) ∑k=0∞S2Zi(k)α(k) =1−(1−fm∗(λc))ρ   +∑k=1∞{1+−θ(1−Λ)[1−(−θq)k]1+θq}α(k) =1−(1−fm∗(λc))ρ+1+θ(q+Λ−1)1+θq   ×∑k=1∞α(k)−θ(Λ−1)1+θq∑k=1∞(−θq)kα(k) =1−(1−fm∗(λc))ρ  +[1+θ(q+Λ−1)][1−fm∗(λc)]2ρ(1+θq)  ×[11−fm∗(λc)]−θ(Λ−1)[1−fm∗(λc)]2ρ(1+θq)  ×[−θq1+θqfm∗(λc)] =1−θ[1−fm∗(λc)](1−Λ)(1+θq)ρ(1+θq)[1+θqfm∗(λc)],

(26) ∑k=0∞S2Z(k)α(k)=1−θ[1−fm∗(λc)]ρ[1+θfm∗(λc)].

## 4. Numerical Results

In this section, the performances of 1*Z*, 2*Z,* and 2*Zi* are investigated through various numerical results for the signaling cost on radio channels. The signaling cost of 1*Z* can be obtained by substituting *θ* = 0  in (24). The performance of 2*Z* is analyzed, using both our proposed model and Lin's model [6], and it can be seen that the results of both models are the same, in every case, as shown in Proposition 3. The performance of 2*Zi* is analyzed, using our proposed model, and is compared with those of 1*Z* and 2*Z*.

We obtain the numerical results, assuming the following environments [2, 6, 7]:

(27) Cp=1,  Cu=4,  θ=0.5,n=8,  λc=1,  λoc=4,  λm=4.

In our examples, the sojourn time in a zone (*t*_*m*_) is assumed to follow an exponential distribution, for convenience. However, since the foregoing equations were derived under the assumption that *t*_*m*_ has a general distribution, any distribution can be assumed.

**Figure 2:**
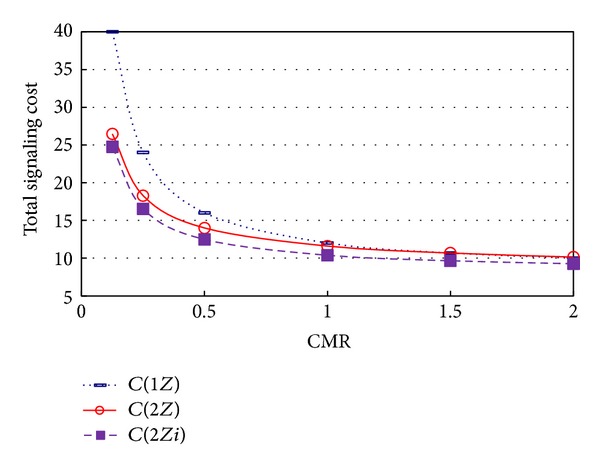
Signaling cost with respect to CMR.

Figure 2 shows the signaling cost with respect to CMR ( = *λ*_*c*_/*λ*_*m*_). It shows the signaling cost when *λ*_*c*_ = 1, with different levels of *λ*_*m*_ from 0.5 to 8.0. The same results are shown in Table 1. As shown in Figure 2 and Table 1, the signaling cost of 2*Z* is lower than that of 1*Z*, in most cases, and the signaling cost of 2*Zi* is lower than that of 2*Z*. Table 1 shows that the signaling cost of 2*Zi* is 22% lower than that of 1*Z* and 10.86% lower than that of 2*Z*, when CMR = 1/2. In fact, the signaling cost of 2*Zi* is lower than those of the other two methods, 2*Z* and 1*Z*, in most cases.

Table 1 also shows that, as CMR increases (*λ*_*m*_ decreases), the signaling cost of 2*Zi* always remains lower than that of 1*Z*, but the reduction ratio of the signaling cost decreases. Conversely, the signaling cost of 2*Zi* is always lower than that of 2*Z*, for all CMR values, but the largest reduction of the signaling cost occurs when CMR = 1/2. Another notable feature of Table 1 is that the signaling cost of 2*Zi* is lower than that of 1*Z*, whereas the signaling cost of 2*Z* is greater than that of 1*Z*, when CMR = 2. When CMR is very large (i.e., *λ*_*m*_ is very small), there are very few location registrations, and 2*Z*, which has an increasing paging cost, may have a disadvantage, compared to 1*Z*. Although it is not shown in Table 1, we can infer that 2*Zi* also may have a disadvantage, compared to 1*Z*, when CMR is very large.

**Figure 3:**
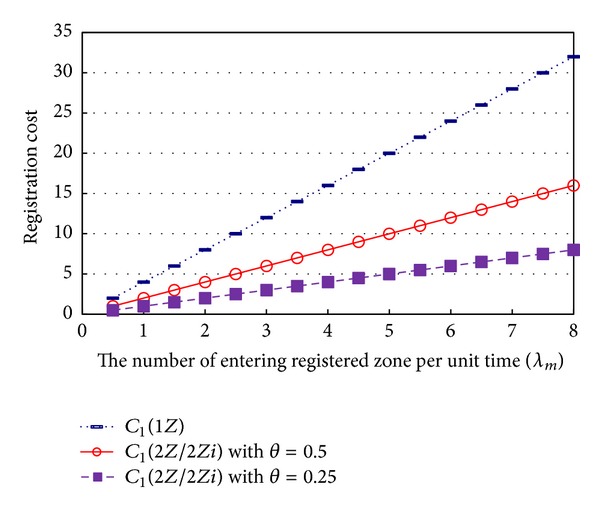
Location registration cost with respect to *λ*_*m*_.

The signaling cost of 2*Zi* and 2*Z* is lower than that of 1*Z*, because 2*Zi* and 2*Z* have lower location registration cost than 1*Z*. To show this feature clearly, we present the location registration cost with respect to *λ*_*m*_, when *λ*_*c*_ = 1, in Figure 3. As shown in Figure 3, the increase of the location registration cost is exactly proportional to the increase of *λ*_*m*_. In addition, the location registration cost is directly related to *θ*, which is the probability of returning to the previous zone. The location registration cost of 2*Z* and 2*Zi* is 25% lower than that of 1*Z*, when *θ* is 0.25, and 50% lower, when *θ* is 0.5.

**Figure 4:**
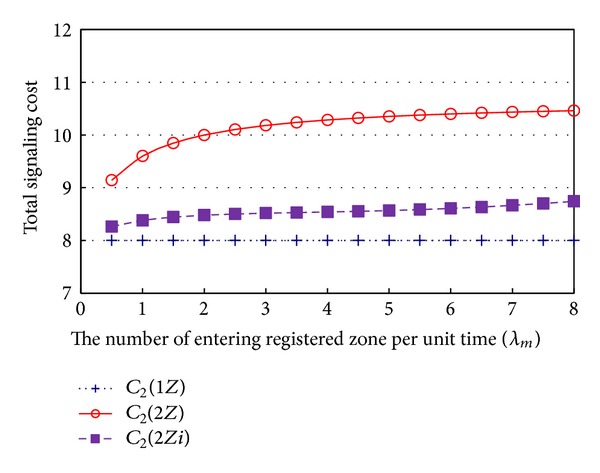
Paging cost with respect to *λ*_*m*_.

The location registration cost of 2*Z* and 2*Zi* is lower, but the paging cost is greater, than that of 1*Z*. To show this feature clearly, we present the paging cost with respect to *λ*_*m*_, when *λ*_*c*_ = 1, in Figure 4.

To show this feature clearly, we present the paging cost with respect to *λ*_*m*_, when *λ*_*c*_ = 1, in Figure 4 and Table 2. As shown in Figure 4 and Table 2, the paging cost of 1*Z* remains constant, while that of 2*Z* and 2*Zi* increases, as *λ*_*m*_ increases. One of the notable results of this study is that, when *λ*_*m*_ = 8, the paging cost of 2*Z*, which is 10.46 (30.77% greater than 8.00 of 1*Z*), can be reduced to 8.74 (9.25% greater than 8.00 of 1*Z*) if 2*Zi* is adopted, which causes the total signaling cost of 2*Zi* to be lower than that of 2*Z*, to an extent corresponding to this reduction, as shown in Table 2.

**Figure 5:**
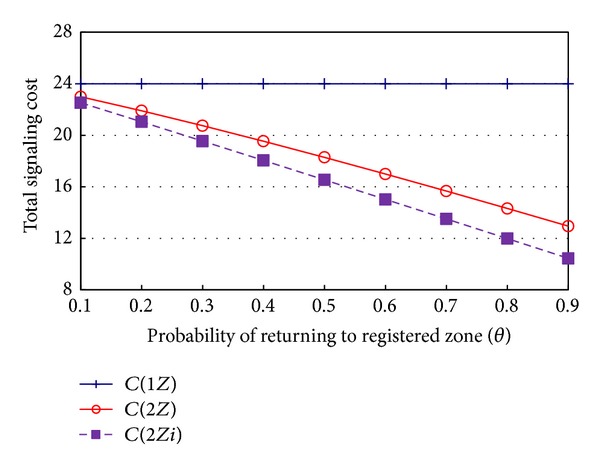
Signaling cost with respect to *θ*.

Figure 5 shows the signaling cost of each method with respect to *θ*, the probability of returning to the previous zone. As shown in Figure 5, the signaling cost of 2*Z* and 2*Zi* decreases, as *θ* increases. In particular, the signaling cost of 2*Zi* decreases more than that of 2*Z*. Even though it is clear that the signaling cost of 2*Z* and 2*Zi* decreases, as *θ* increases, it seems to be unreasonable to assume that *θ* is larger than 0.5, in a real-world mobile communication environment.

**Figure 6:**
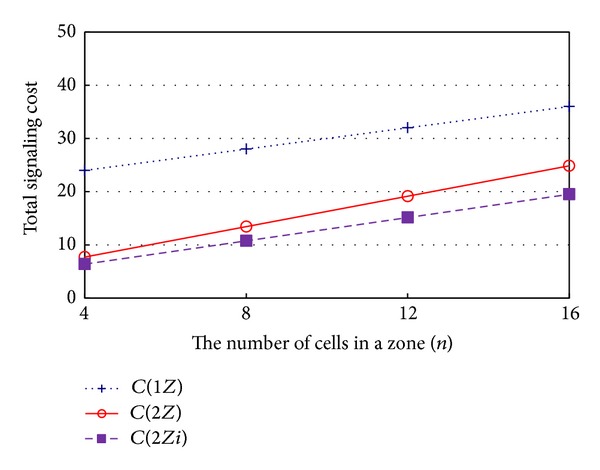
Signaling cost with respect to the number of cells in a zone (*λ*_*oc*_ = *λ*_*m*_ = 5).

Finally, Figure 6 shows the signaling cost with respect to *n*, the number of cells in a zone. In this case, since the location registration cost remains constant, the overall amount of the signaling cost will increase, as the number of cells in a zone increases, due to the increase of the paging cost. As shown in Figure 6, the signaling costs of 1*Z*, 2*Z,* and 2*Zi* all increase, as the number of cells in a zone increases, but the increased ratios of 2*Zi* and 1*Z* are lower than that of 2*Z*. That is, if the other conditions are the same, 2*Z* is more superior to 1*Z*, and 2*Z* is more superior to 2*Zi*, respectively, as the paging cost increases.

## 5. Conclusion

Many efficient mobility management methods have been suggested, to minimize the signaling cost on radio channels. This study considered the zone-based registration methods that are widely used in the majority of mobile communication networks.

We provided a new mathematical model to analyze the performance of the zone-based registration methods, 2*Z* and 2*Zi*, by considering implicit registration effects of outgoing calls from a mobile, which were not considered properly in the previous studies. It should be noted that our mathematical model is simple, compared to the previous studies, but provides the exact performance of 2*Zi* for the first time. Also, our model can easily be applied to 2*Z* and 1*Z* and provides the same results as Lin's previous study on 2*Z* and 1*Z*. From various numerical results by using our model, we showed that 2*Zi* is superior to 2*Z* as well as 1*Z*, in most cases.

Our results are helpful in considering which registration scheme should be adopted. For further study, we will consider the case where a mobile can have multiple zones, to get the performance of every type of zone-based registration.

## Figures and Tables

**Figure 7:**
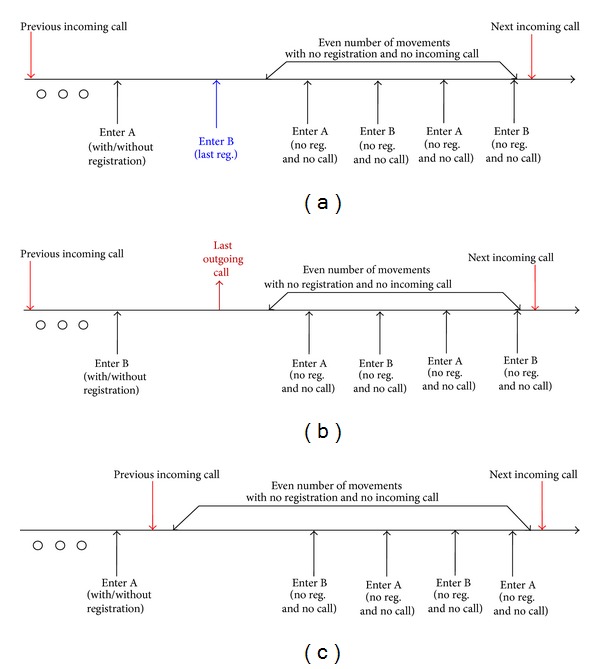
Situations when the system has the correct view of the latest visited zone. (a) Case 1: the last registration is followed by an even number of movements. (b) Case 2: the last outgoing call is followed by an even number of movements with no registration and no outgoing call. (b) Case 3: there are an even number of movements with no registration and no outgoing call.

**Table 1:** Signaling cost with respect to CMR.

CMR (=*λ*_*c*_/*λ*_*m*_)	0.125	0.25	0.5	1	1.5	2
*C*(1*Z*)	40.00	24.00	16.00	12.00	10.68	10.00
*C*(2*Z*)	26.46	18.29	14.00	11.60	10.68	10.14
*C*(2*Zi*)	24.74	16.54	12.48	10.38	9.65	9.26
Reduction ratio (%) =100 × [1 − *C*(2*Zi*)/*C*(1*Z*)]	38.15	31.08	22.00	13.49	9.63	7.38
Reduction ratio (%) =100 × [1 − *C*(2*Zi*)/*C*(2*Z*)]	6.50	9.54	10.86	10.51	9.60	8.69

**Table 2:** Paging cost with respect to *λ*_*m*_.

*λ*_*m*_	0.5	1	2	4	8
*C*_2_(1*Z*)	8.00	8.00	8.00	8.00	8.00
*C*_2_(2*Z*)	9.14	9.60	10.00	10.29	10.46
*C*_2_(2*Zi*)	8.26	8.38	8.48	8.54	8.74
Reduction ratio (%) =100 × [1 − *C*_2_(2*Zi*)/*C*_2_(1*Z*)]	9.64	12.70	15.20	16.97	16.44
Reduction ratio (%) =100 × [*C*_2_(2*Z*)/*C*_2_(1*Z*) − 1]	14.29	20.00	25.00	28.57	30.77
Reduction ratio (%) =100 × [*C*_2_(2*Zi*)/*C*_2_(1*Z*) − 1]	3.27	4.76	6.00	6.76	9.27

## Appendix

### Derivation of *ω*(*k*)

Proposition 6 The probability that the first paging succeeds in 2Zi, given that the mobile moves across *k* zones between two incoming calls, is composed of three probabilities for three exclusive cases, *ω*_1_(*k*), *ω*_2_(*k*),   and  *ω*_3_(*k*), and their sum, *ω*(*k*),   is given by

(A.1) ω(k) ={(1−θ)(1−Λ)[1−θk+1qk+1]1−θ2q2 +  p(1−Λ)θ2q[1−θk−1qk−1]1−θ2q2+Λ,    if  k  is odd number  (k≥3),(1−θ)(1−Λ)[1−θkqk]1−θ2q2+θkqk(1−Λ) +  p(1−Λ)θ2q[1−θkqk]1−θ2q2+Λ,    if  k  is even number  (k≥2),(1−θ)(1−Λ)+Λ=1−θ+θΛ,    if  k=1,1,   if  k=0.

Proof If the probability Pr[zone  hit  in  2*Zi*]  that the first paging succeeds in 2*Zi* is composed of the three probabilities, *ω*_1_,  *ω*_2_,   and  *ω*_3_, for three exclusive cases, then we have

(A.2) $$\begin{array}{c} \text{Pr}\left[\text{zone hit in}\;2Zi\right]=\omega_{1}+\omega_{2}+\omega_{3}. \end{array}$$

Each probability for the three exclusive cases can be derived as follows. (i) Case 1. If, between two incoming calls, the last registration is followed by an even number of movements with no registration and no outgoing call, then the first paging succeeds (see Figure 7(a)). Letting *ω*_1_ be the probability of Case 1, we have

(A.3) $$\begin{array}{c} \omega_{1}=\overset{\infty }{\underset{k=1}{\sum }}\omega_{1}\left(k\right)\alpha \left(k\right). \end{array}$$

In the above, *ω*_1_(*k*) is the conditional probability that, given that the mobile moves across *k* zones between two incoming calls, the last registration is followed by an even number of movements, with no registration and no outgoing call. This can be derived by

(A.4) ω1(k) ={(1−θ)(1−Λ)[1−θkqk]1−θ2q2,if  k  is even number,(1−θ)(1−Λ)[1−θk−1qk−1]1−θ2q2,if  k  is odd number,

since, for *k* ≥ 1,

(A.5) ω1(k)=(1−θ)∑i=0⌊(k−1)/2⌋θ2i(1−P[toc<tm])2iP[tc<toc]=(1−θ)∑i=0⌊(k−1)/2⌋θ2iq2i(1−Λ)=(1−θ)(1−Λ)[1−θ2⌊(k−1)/2⌋+2q2⌊(k−1)/2⌋+2]1−θ2q2.

(ii) Case 2. If, between two incoming calls, the last outgoing call is followed by an even number of movements, with no registration and no further outgoing calls, then the first paging succeeds (see Figure 7(b)). Letting *ω*_2_ be the probability of Case 2, we have

(A.6) $$\begin{array}{c} \omega_{2}=\overset{\infty }{\underset{k=1}{\sum }}\omega_{2}\left(k\right)\alpha \left(k\right). \end{array}$$

In the above, *ω*_2_(*k*) is the conditional probability that, given that the mobile moves across *k* zones between two incoming calls, the last outgoing call is followed by an even number of movements, with no registration and no further outgoing call. This can be derived by

(A.7) ω2(k) ={Λ+p(1−Λ)θ2q[1−θkqk]1−θ2q2,    if  k  is even number,Λ+p(1−Λ)θ2q[1−θk−1qk−1]1−θ2q2,    if  k  is odd number  (k≥3),Λ,   if  k=1,

since, for *k* ≥ 2,

(A.8) ω2(k)=P[tc>toc]+P[tm>toc] ×∑i=1⌊k/2⌋θ2i(1−P[toc<tm])2i−1P[tc<toc]=Λ+p∑i=1⌊k/2⌋θ2iq2i−1(1−Λ)=Λ+p(1−Λ)θ2q[1−θ2⌊k/2⌋q2⌊k/2⌋]1−θ2q2.

(iii) Case 3. If, between two incoming calls, there are an even number of movements with no registration and no outgoing call, then the first paging succeeds (see Figure 7(c)). Letting *ω*_3_ be the probability of Case 3, we have

(A.9) $$\begin{array}{c} \omega_{3}=\overset{\infty }{\underset{k=1}{\sum }}\omega_{3}\left(k\right)\alpha \left(k\right). \end{array}$$

In the above, *ω*_3_(*k*) is the conditional probability that, given that the mobile moves across *k* zones between two incoming calls, there are an odd number of movements, with no registration and no outgoing call. This can be derived by

(A.10) $$\begin{array}{c} \omega_{3}\left(k\right)=\{\begin{array}{cc} \theta^{k}q^{k}\left(1-\Lambda \right), & \text{if}\;\;k\;\;\text{is even number},\\ 0, & \text{if}\;\;k\;\;\text{is odd number}, \end{array} \end{array}$$

since, for even number *k*,

(A.11) ω3(k)=∑i=1k/2θ2i(1−P[toc<tm])2iP[tc<toc]=θkqk(1−Λ).

Finally, we have

(A.12) Pr[zone  hit  in  2Zi]=ω1+ω2+ω3=∑k=1∞ω1(k)α(k)+∑k=1∞ω2(k)α(k) +∑k=1∞ω3(k)α(k)=∑k=1∞ω(k)α(k),

where

(A.13) ω(k) ={(1−θ)(1−Λ)[1−θk+1qk+1]1−θ2q2 +  p(1−Λ)θ2q[1−θk−1qk−1]1−θ2q2+Λ,    if  k  is odd number  (k≥3),(1−θ)(1−Λ)[1−θkqk]1−θ2q2+θkqk(1−Λ) +  p(1−Λ)θ2q[1−θkqk]1−θ2q2+Λ,    if  k  is even number  (k≥2),(1−θ)(1−Λ)+Λ=1−θ+θΛ,    if  k=1,1,   if  k=0.

* *
